# MUC4 activates HER2 signalling and enhances the motility of human ovarian cancer cells

**DOI:** 10.1038/sj.bjc.6604517

**Published:** 2008-07-29

**Authors:** M P Ponnusamy, A P Singh, M Jain, S Chakraborty, N Moniaux, S K Batra

**Affiliations:** 1 Department of Biochemistry and Molecular Biology, University of Nebraska Medical Center, Omaha, NE 68198-5870, USA; 2 Eppley Institute for Research in Cancer and Allied Diseases, University of Nebraska Medical Center, Omaha, NE 68198-5870, USA

**Keywords:** MUC4, ovarian cancer, HER2, motility

## Abstract

The mucin MUC4 is a high molecular weight transmembrane glycoprotein. It consists of a mucin-type subunit (MUC4*α*) and a transmembrane growth factor-like subunit (MUC4*β*). The mucin MUC4 is overexpressed in many epithelial malignancies including ovarian cancer, suggesting a possible role in the pathogenesis of these cancers. In this study, we investigated the functional role of MUC4 in the human ovarian cancer cell line SKOV3. The mucin MUC4 was ectopically expressed by stable transfection, and its expression was examined by western blot and confocal microscopy analyses. The *in vitro* studies demonstrated an enhanced motility of MUC4-expressing SKOV3 cells compared with the vector-transfected cells. The mucin MUC4 expression was associated with apparent changes in actin organisation, leading to the formation of microspike, lammelopodia and filopodia-like cellular projections. An enhanced protein expression and activation of HER2, a receptor tyrosine kinase, was also seen, although no significant change was observed in HER-2 transcript levels in the MUC4-transfected SKOV3 cells. Reciprocal co-immunoprecipitation revealed an interaction of MUC4 with HER2. Further, the MUC4-overexpressing SKOV3 cells exhibited an increase in the phosphorylation of focal adhesion kinase (FAK), Akt and ERK, downstream effectors of HER2. Taken together, our findings demonstrate that MUC4 plays a role in ovarian cancer cell motility, in part, by altering actin arrangement and potentiating HER2 downstream signalling in these cells.

Ovarian cancer is a highly lethal disease. It often goes undiagnosed until the last stage when it has already spread to distant sites. The current therapeutic strategies for tackling this malignancy are still inefficient and tumour recurrence of up to 70% is observed in patients with advanced ovarian carcinoma even after treatment (Auersperg *et al*, 2001, 2002; Jemal *et al*, 2007). The mortality from ovarian cancer could be significantly lowered by developing new strategies for the early diagnosis and treatment. A better understanding of the molecular mechanisms underlying its progression and the aggressive phenotype is therefore an urgent requirement for developing novel and effective therapeutic modalities for the treatment of this deadly malignancy.

Mucins are large, heavily glycosylated proteins that provide protection to the luminal epithelial surfaces under normal physiological conditions (Moniaux *et al*, 2001; Hollingsworth and Swanson, 2004). Alteration in the expression or glycosylation pattern of mucins is often associated with the development of cancer by influencing key cellular functions including growth, differentiation, transformation, adhesion and invasiveness of tumour cells and immune surveillance (Andrianifahanana *et al*, 2001; Hollingsworth and Swanson, 2004). The mucin MUC4 is a transmembrane mucin that frequently displays an altered expression in many cancers (Carraway *et al*, 2001b; Hollingsworth and Swanson, 2004; Andrianifahanana *et al*, 2006).

Previous studies from our laboratory have revealed an aberrant expression of the MUC4 in more than 90% of ovarian tumours, whereas a very low to no expression was detected in the normal ovaries (Chauhan *et al*, 2006). An overexpression of MUC4 mRNA has also been previously reported in ovarian cancer (Giuntoli *et al*, 1998; Lopez-Ferrer *et al*, 2001). The deduced full-length amino-acid sequence of the MUC4 apoprotein shows the presence of a leader peptide, a serine and threonine-rich non-tandem repeat region, a central large tandem repeat domain containing 16-amino-acid repetitive units, regions rich in potential *N*-glycosylation sites, two cysteine-rich domains, a putative GDPH proteolytic cleavage site, three epidermal growth factor (EGF)-like domains, a hydrophobic transmembrane domain and a short cytoplasmic tail. The MUC4 mucin is considered to be a homologue of the rat sialo-mucin complex (SMC, rat MUC4) because of similarities in their structural organisation (Komatsu *et al*, 1999; Moniaux *et al*, 1999). The SMC is also a heterodimeric glycoprotein composed of an *O*-glycosylated mucin subunit ASGP-1, and an *N*-glycosylated transmembrane subunit ASGP-2 containing two EGF domains (Komatsu *et al*, 2000). The mucin MUC4 also possesses two subunits: an extracellular mucin-like subunit, MUC4, and a growth factor-like transmembrane subunit, MUC4*β*, containing three EGF-like domains (Moniaux *et al*, 1999, 2001). The overexpression of SMC is associated with the suppression of both cell adhesion and immune killing of tumour cells by altering cell-surface properties and promoting tumour growth *in vitro* or *in vivo* (Komatsu *et al*, 1999, 2000). The SMC acts as a ligand for the receptor tyrosine kinase ErbB2/HER2/neu, via one of its two EGF-like domains, and induces its phosphorylation (Komatsu *et al*, 2001; Carraway *et al*, 2001b, 2002a, 2002b). Similarly, MUC4 has been shown to be involved in the growth and metastasis of pancreatic cancer cells (Singh *et al*, 2004; Moniaux *et al*, 2007). In pancreatic cancer cells, MUC4 induces ultra-structural alterations and promotes tumour cell proliferation and survival and interferes in the interaction of tumour cells with extracellular matrix (Chaturvedi *et al*, 2007; Moniaux *et al*, 2007).

In this study, we have investigated the functional consequences of ectopic MUC4 expression in a human ovarian cancer cell line SKOV3. Our studies demonstrate a direct association of the MUC4 mucin with increased motility in ovarian cancer cells. We also show that MUC4 interacts with HER2, enhances its expression and potentiates its downstream signalling.

## Materials and methods

### Cell culture and stable transfection of MUC4-expression construct

The MUC4 cDNA is very large (29 kb); therefore, it was difficult to clone the entire coding sequence into any available expression vector. Thus, we reduced the size of the repetitive sequence (by decreasing the number of tandem repeats) and generated an MUC4-expression plasmid, which encodes a protein of 320 kDa (Moniaux *et al*, 2007). The SKOV3 ovarian cancer cells were procured from ATCC (Manassas, VA, USA) and cultured in DMEM medium supplemented with 10% foetal calf serum and antibiotics. The cultures were maintained in a humidified atmosphere at 37 °C with 5% CO_2_. The MUC4-expression plasmid and empty vector (pSecTag/MUC4 and pSegTag) were stably transfected in SKOV3 ovarian cancer cells by Fugene (Invitrogen Corp., Carlsbad, CA, USA), following the manufacturer's protocol. The Zeocin-resistant colonies were isolated by the ring cloning method and maintained in medium supplemented with 250 *μ*g ml^−1^ Zeocin (Invitrogen Corp.). Medium was replaced with complete medium without antibiotic supplement at least 5 days before any analysis.

### RNA isolation and quantitative RT–PCR for HER2 mRNA

Transcript levels of HER2 in MUC4-transfected SKOV3 cells were measured by SYBR® Premix *Ex Taq*™ kit. cDNA was synthesised using 2 *μ*g of total RNA, oligo(dT)_18_ primer and Superscript RT (Invitrogen Corp.). Quantitative real-time PCR was performed using 1 *μ*l of a 1 : 5 dilution of first-strand cDNA using the SYBR Premix *Ex Taq* kit (Takara Bio, Madison, WI, USA) and primers specific to HER2 and RPL13A on an ABI 7500 Sequence Detection System (Applied Biosystems, Foster, CA, USA). The receptor tyrosine kinase HER2 was amplified using the forward primer 5′-TCA CCT ACA ACA CAG ACA CGT TTG-3′ and the reverse primer 5′-ATC CCA CGT CCG TAG AAG GTA-3′, whereas RPL13A was amplified using the forward primer 5′-ATC GTG GCT AAA CAG GTA CTG-3′ and the reverse primer 5′-GCA CGA CCT TGA GGG CAG C-3′. The efficiency of the PCR and the specificity of primers were determined using serial dilutions of template cDNA and dissociation curve analysis. Each cDNA sample was used in triplicate, and a reaction without any cDNA was used as negative control. The levels of HER2 mRNA were then normalised to the RPL13A mRNA levels, and results were graphically represented as a fold difference in HER2 mRNA level in MUC4-overexpressing versus empty-vector-transfected cells.

### Immunoprecipitation and immunoblot analysis

Cells were grown to 70–80% confluence in a 5% CO_2_ incubator at 37 °C. Cells were rinsed once with ice-cold phosphate-buffered saline (PBS) containing 1 mM sodium orthovanadate and lysed in Triton X-100 lysis buffer (150 mM NaCl, 50 mM Tris–HCl [pH 8.0], 5 mM NaF, 1.0% Triton X-100, 1 mM sodium orthovanadate, 5 *μ*g of aprotinin per ml and 5 *μ*g of leupeptin per ml) for 25–35 min at 4 °C. The lysates were centrifuged at 16 000 **g** for 20 min at 4 °C. Protein concentrations were measured using the micro-BCA protein estimation kit (Bio-Rad, Hercules, CA, USA). Equal amounts of protein were incubated overnight with anti-MUC4 (mouse monoclonal) or anti-HER2 (Rabbit polyclonal) antibodies in a 500-*μ*l total volume. Protein A+ G-Sepharose beads (Oncogene Research, Boston, MA, USA) were added to the lysate–antibody mix and incubated on a rotating platform for 3 h at 4 °C and washed four times with lysis buffer. The immunoprecipitates or total cell lysates were electrophoretically resolved on a gel. Sodium dodecyl sulphate–agarose (2%) gel electrophoresis was performed for MUC4 using 20 *μ*g protein samples under reducing conditions. For HER2, FAK, Akt, ERK, p38 (rabbit polyclonal) and *β*-actin (mouse monoclonal) expression, SDS–PAGE (10%) was performed under similar conditions. Resolved proteins were transferred on to the PVDF membrane. After quick washing in PBST (PBS and 0.1% Tween 20), the membranes were blocked in 5% non-fat dry milk in PBS for at least 2 h and then incubated with primary antibodies (anti-MUC4, both activated form and total of HER2, FAK, Akt, ERK, p38 and *β*-actin). The immunoblots were washed five times (5 × 10 min), incubated with anti-mouse (for MUC4 and *β*-actin blots) and anti-rabbit (for HER2, FAK, Akt, ERK and p38 blots) horseradish peroxidase-conjugated secondary antibodies, washed five times (5 × 10 min), reacted with enhanced chemiluminescence ECL reagent (Amarsham Bioscience, Buckinghamshire, UK) and exposed to X-ray film to detect the signal.

### Confocal immunofluorescence microscopy

Derived cells and SKOV3 were seeded at low density on sterilised cover slips and allowed to grow for 20 h. Cells were washed with Hanks buffer containing 0.1 M HEPES, and fixed in ice-cold methanol at −20 °C for 2 min and blocked with 10% goat serum (Jackson Immunoresearch Labs Inc., West Grove, PA, USA) containing 0.05% Tween-20 for at least 30 min. For Phalloidin staining, cells were fixed in 3.6% formaldehyde–PBS solution and permeabilised with 0.1% TritonX-100 in PBS for 20 min at room temperature. After the blocking step and a quick wash in PBS, cells were incubated with the anti-MUC4 monoclonal antibody, and with anti-HER2 polyclonal antibodies for 60 min at room temperature. Cells were then washed (4 × 5 min) with PBS containing 0.05% Tween-20 (PBST) and then incubated with FITC-conjugated anti-mouse and Texas red-conjugated anti-rabbit secondary antibodies (Jackson Immunoresearch Labs Inc., West Grove, PA, USA) for 30 min at room temperature in the dark. Cells were washed (5 × 5 min) again and mounted on glass slides in anti-fade Vectashield mounting medium (Vector Laboratories, Burlingame, CA, USA). Laser confocal microscopy was performed by using an LSM 510 microscope (Carl Zeiss GmbH, Thornwood, NY, USA). Photographs were taken in each channel separately and digitally merged for co-localisation studies.

### Motility assay

For motility assays, 0.5 × 10^6^ cells were plated in the top chamber of monocoated polyethylene teraphthalate membranes (six-well insert, pore size 8 *μ*m) (Becton Dickinson, San Jose, CA, USA). The cells were incubated for 24 h, and the cells that did not migrate through the pores in the membrane were removed by scraping the membrane with a cotton swab. The migrated cells in the membrane were stained with a diff-Quick cell stain kit, and cells in 10 random fields of view at × 10 magnification were counted and expressed as the average number of cells per field of view. Three independent experiments were performed in each case. The data were represented as the average of the three independent experiments with the standard deviation indicated.

## Results

### Ectopic expression of the MUC4 mucin in transfectants

**Figure 1:**
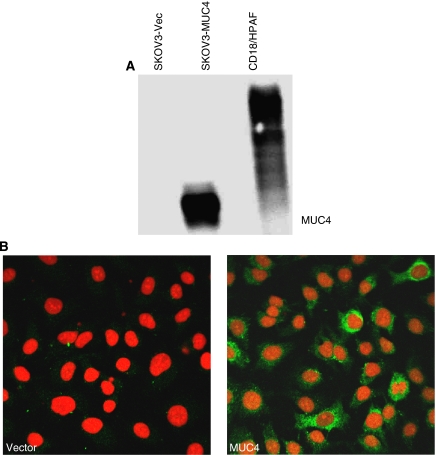
Expression profile of MUC4 in MUC4-transfected SKOV3 cells. (**A**) Western blot analysis of MUC4 expression in the vector-transfected (SKOV3-Vec) and MUC4-transfected (SKOV3-MUC4) SKOV3 cell line. The MUC4-expressing pancreatic cancer cell line CD18/HPAF was used as a positive control. A total of 20 *μ*g protein from cell extracts was resolved by electrophoresis on a 2% SDS-agarose gel, transferred to the PVDF membrane and probed with anti-MUC4 mouse monoclonal antibody (8G7). The membrane was then incubated with horseradish peroxidase-labeled goat anti-mouse immunoglobulin. The signal was detected using an electrochemiluminescence reagent kit. The MUC4 mucin is a high molecular weight glycoprotein and the predicted size of the transfected MUC4 protein is 320 kDa. (**B**) Localisation of MUC4 by confocal microscopy. Cells were grown at low density on sterilised cover slips, washed and fixed in ice-cold methanol at −20 °C. After blocking with 10% goat serum, cells were incubated with the anti-MUC4 mouse monoclonal antibody, washed and followed by incubation with FITC-conjugated goat anti-mouse IgG (× 630 original magnification).

We have screened a large number of human ovarian cancer cell lines for expression of MUC4. Our recent study showed that MUC4 is expressed in above 95% of ovarian tumours (Chauhan *et al*, 2006); however, it was not detected in the majority of cell lines that were screened (SKOV3, OVCAR3, OVCAR5, OVCAR8, A2780, COV362.4, COV 664, COV434, KGN, SB247, etc.). To investigate the function of MUC4, the cDNA of MUC4 was stably expressed in SKOV3 cells and transfectants were analysed by immunoblotting (8G7 antibody) and confocal microscopy of the immunostained cells. Expression of MUC4 was observed in the MUC4-transfected pooled population of cells (SKOV3-MUC4) and not in the vector control (SKOV3-Vec) (Figure 1A). CD18/HPAF is a MUC4-expressing pancreatic cancer cell line used as a positive control (Figure 1A). Immunofluorescence confocal microscopy showed the expression and localisation of MUC4 in both the plasma membrane and cytoplasm in more than 90% of the MUC4-transfected cells, whereas the vector-transfected control cells showed no MUC4 staining (Figure 1B). Propedium iodide was used as a nuclear stain.

### MUC4 expression enhances the motility of SKOV3 cells and causes a rearrangement of the actin cytoskeleton

**Figure 2:**
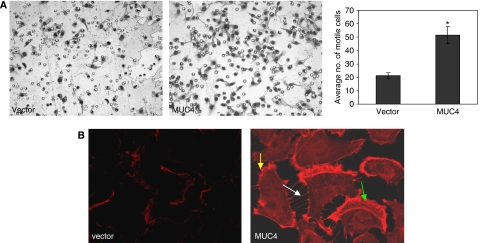
Effect of MUC4 overexpression on the motility and actin organisation of SKOV3 cells. (**A**) Cells (0.5 × 10^6^) were plated on non-coated membranes for the cellular motility assays. The cells were incubated for 24 h and allowed to move under a chemotactic effect. The cells that did not migrate through the pores in the membrane were removed by scraping the membrane with a cotton swab, whereas the cells that migrated through the pores were stained. The number of cells traversing the membrane was determined by averaging ten random fields of view at × 10. The data are expressed as the average number of cells per fields of view and is the average of three independent experiments. Cell motility was significantly (*P*<0.05) increased in the MUC4-transfected SKOV3 cells. (**B**) Cells were seeded on cover slips and fixed with 3.7% formaldehyde and permeabilised with 0.1% Triton X-100. Cells were stained with 50 *μ*g ml^−1^ fluorescent Phalloidin conjugate solution. SKOV3 cells transfected by MUC4 were associated with the presence of more microspikes (yellow arrows), lammelopodia (green arrows) and filopodia (white arrows)-like cellular projections with dense actin specifically at the cellular protrusions compared with the empty vector control cells (original magnification 1000 × ).

We analysed the cellular motility in both vector-transfected and MUC4-overexpressing cells by using Boyden's chamber assay. The MUC4-transfected cells showed a significant (*P*<0.05) increase in motility when compared with the vector-transfected cells (Figure 2A). Cell motility is typically associated with the coordinated disassembly and reformation of the cortical actin network (Cunningham *et al*, 1991, 1992). Polymerisation of globular actin (G-actin) leads to the formation of long fibrous molecules called filamentous actin (F-actin). The localisation and distribution of F-actin was analysed by Phalloidin staining in both MUC4-overexpressing and vector-transfected SKOV3 cells. Confocal microscopy of the Phalloidin-stained cells showed filopodia (white arrow), lammelopodia (green arrow) and microspikes (yellow arrow) in MUC4-transfected SKOV3 cells (Figure 2B). In contrast, the vector-transfected SKOV3 cells showed less staining in the cytoplasm and did not show any cellular outgrowth (Figure 2B). Presence of structures like lammelopodia, filopodia and microspikes may be a cause of increased motility in the MUC4-transfected cells.

### MUC4 interacts with HER2 and increases its expression in SKOV3 cells

**Figure 3:**
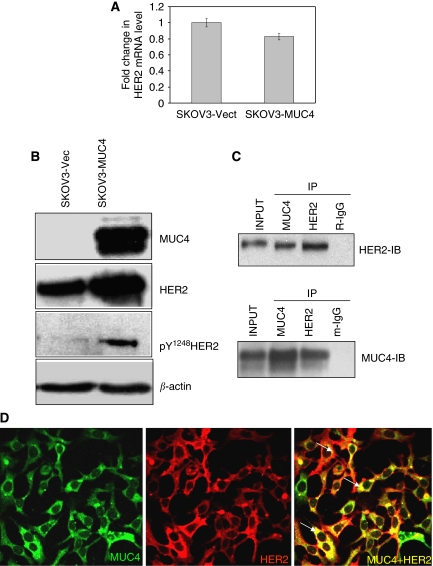
The mucin MUC4 interacts with HER2 and induces the expression of HER2. (**A**) Estimation of HER2 mRNA levels in MUC4-transfected SKOV3 ovarian cancer cells by real-time RT–PCR. A total of 2 *μ*g of RNA was reverse-transcribed and diluted to a total volume of 100 *μ*l (5 × dilution). A total of 1 *μ*l of this cDNA was used in each reaction in a total reaction volume of 10 *μ*l. Each reaction was plated in triplicate. The results are expressed as the fold changes of HER2 in MUC4-expressed SKOV3 cells relative to that in the vector-transfected cells. No significant change was observed in HER2 transcript levels compared with empty vector-transfected cells (*P*-values by applying the *t*-test: 0.889). (**B**) Western blot analysis for HER2 expression and HER2 tyrosine phosphorylation in SKOV3-derived cell lines. A total of 20 *μ*g of protein from the cell lysate was resolved by SDS–PAGE, transferred to a PVDF membrane and probed with antibodies against MUC4, HER2, phosphor-Tyr^1248^ HER2 and *β*-actin. SKOV3 cells transfected by MUC4 showed an increased expression and tyrosine phosphorylation of HER2 at the Tyr^1248^ residue. No phosphorylation of HER2 was seen in the SKOV3-vector control. (**C**) Reciprocal co-immunoprecipitation analysis to show the interactions between MUC4 and HER2. Lysates from the MUC4-overexpressing SKOV3 cell lines were utilised for immunoprecipitation with MUC4 and HER2 antibodies. The immunoprecipitates were electrophoretically resolved on 2% SDS-agarose (for MUC4) and 10% polyacrylamide gel (for HER2), and immunoblotted with anti-MUC4 or anti-HER2 antibodies. The isotype antibodies were used as immunoprecipitation controls. (**D**) Co-localisation of MUC4 and HER2 in SKOV3 MUC4-transfected cells. Cells were grown at low density on sterilised cover slips, washed and fixed in ice-cold methanol at −20 °C. After blocking with 10% goat serum, cells were incubated with the anti-MUC4 mouse monoclonal antibody and anti-HER2 polyclonal antibody followed by incubation with FITC-conjugated goat anti-mouse IgG (× 630 original magnification). The green staining shows MUC4 expression, red staining shows HER2 expression and the yellow staining shows the co-localisation.

Our previous studies have shown that the knockdown of MUC4 expression significantly reduces the expression level and phosphorylation status of HER2 in the CD18/HPAF pancreatic cell line (Singh *et al*, 2004). The overexpression of HER2 has also been correlated with increased cellular dynamics and motility in breast cancer cells (Grothey *et al*, 2000). The expression of HER2 transcript was measured by quantitative real-time reverse-transcription assay in MUC4-transfected SKOV3 cells. There was no significant variation in the HER2 transcript level (*P*-values by applying the *t*-test: 0.889) (Figure 3A). Western blot analysis of HER2 in MUC4-transfected SKOV3 cells showed an increased expression of HER2 and its activated form, pY^1248^HER2, as compared with the vector-transfected cells (Figure 3B). Furthermore, our reciprocal co-immunoprecipitation studies indicated that MUC4 and HER2 interact with each other in the MUC4-transfected cells (Figure 3C). Confocal microscopy and digital merging of the MUC4- and HER2-immunostained cells demonstrated significant colocalisation of both the proteins in the MUC4-transfected SKOV3 cells (Figure 3D).

### Activation of HER2 downstream cell signalling in MUC4-transfected SKOV3 cells

**Figure 4:**
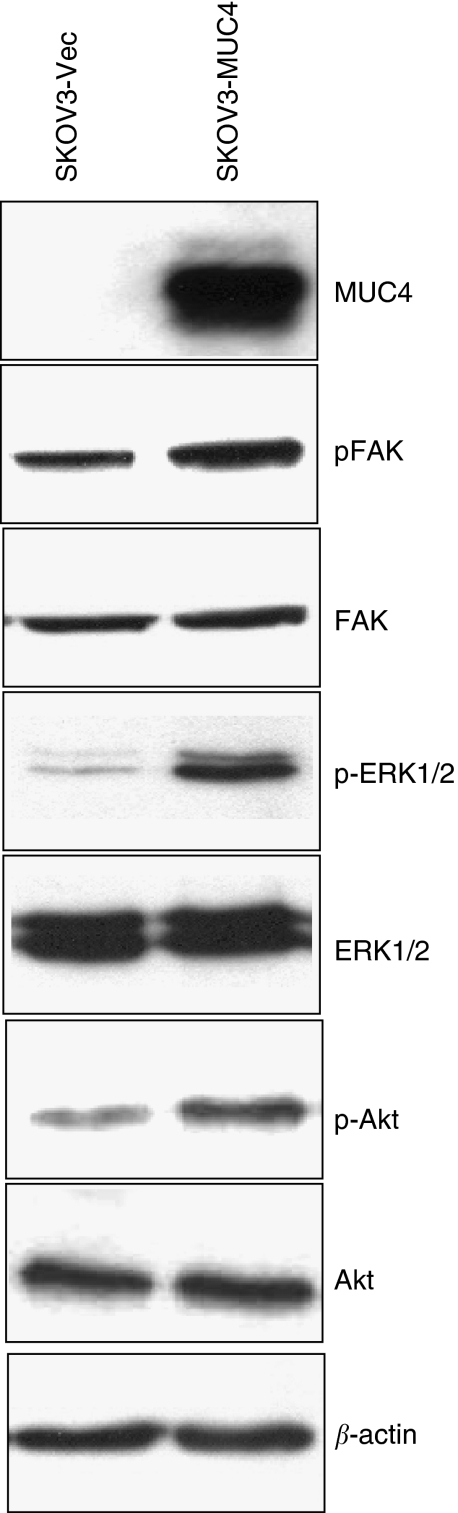
The mucin MUC4 induces HER2 downstream cell signalling in SKOV3 cells. A total of 20 *μ*g of cell lysates from SKOV3-vector and SKOV3-MUC4-transfected cells were used for immunoblotting with MUC4, FAK, phosphorylated FAK, ERK, phosphorylated ERK, Akt and phosphorylated Akt antibodies. For MUC4 detection, proteins were resolved on 2% SDS-agarose gel; other proteins were resolved on 10% SDS-polyacrylamide gel. *β*-actin was used as an internal control. SKOV3 cells transfected by MUC4 clearly showed increased activation of FAK, Akt and ERK in MUC4-transfected SKOV3 cells, whereas the vector-transfected cells showed a lesser activation of FAK, Akt and ERK. The levels of total FAK, Akt and ERK remain unchanged.

Downstream signalling of HER2 promotes survival and motility of cancer cells (Gabarra-Niecko *et al*, 2003; Mitra and Schlaepfer, 2006). Focal adhesion kinase is linked to the downregulation of HER2 activity at the cellular periphery, which appears to be important for the formation of motile cells (Mitra and Schlaepfer, 2006). Focal adhesion kinase is a non-receptor tyrosine kinase localised to focal adhesions (Schaller *et al*, 1992). It is phosphorylated in response to a number of stimuli including the clustering of integrins (Kornberg *et al*, 1991). Focal adhesion kinase is overexpressed in invasive and metastatic tumours (Owens *et al*, 1996). An increase level of activated FAK (pY925) was observed in MUC4-transfected SKOV3 cells when compared with the vector-transfected cells (Figure 4). The levels of total FAK, however, remained unchanged in both MUC4- and vector-transfected cells (Figure 4), suggesting that MUC4 activates FAK, either by a direct or an indirect mechanism, which may be responsible to the greater motility observed in the MUC4-transfected cells.

The effect of MUC4 on signalling through the AKT and ERK and p38 pathways was also examined in both SKOV3 sublines. The ERK1/2 and Akt signalling pathways play a major role in the migration of cancer cells, respectively. The MUC4-transfected SKOV3 cells showed a significant increase in the levels of activated ERK1/2 and Akt (Figure 4) compared with the vector-transfected cells (Figure 4). The total levels of ERK1/2 and Akt, however, remained unchanged (Figure 4). The increased activation of ERK1/2 and Akt may be due to the interaction of the ectopically expressed MUC4 with HER2. The expression level of phospho p38 and total p38 did not show any variation in both the MUC4 and empty vector-transfected cells (data not shown).

## Discussion

The mucin MUC4 is aberrantly expressed in several epithelial malignancies. Our recent studies revealed an overexpression of MUC4 in ovarian (Chauhan *et al*, 2006) and pancreatic tumours (Andrianifahanana *et al*, 2001), whereas its expression was downregulated in prostate cancer (Singh *et al*, 2006). Other studies from our lab have shown that MUC4 is involved in pancreatic tumour cell growth and metastasis (Singh *et al*, 2004; Chaturvedi *et al*, 2007). In this study, we have provided experimental evidence that MUC4 interacts with HER2, potentiates its downstream signalling and enhances the motility of ovarian cancer cells. Our findings provide experimental support for the hypothesis that MUC4 expression is associated with a higher metastatic potential and thereby a poor prognosis in ovarian cancer.

One of the important observations of this study was the interaction of MUC4 and HER2 in ovarian cancer cells, which was also associated with the potentiation of HER2 downstream signalling. Normally, epithelial cells have polarised morphology with apical, basal and lateral regions. The mucin MUC4, wherever present in normal cells, has an apical localisation. However, during the course of malignant transformation, cells lose their polarity, which may allow MUC4 to form novel protein–protein interactions (such as between MUC4 and HER2) with proteins that are normally localised to the basal or lateral surfaces (Ramsauer *et al*, 2003, 2006). In fact, MUC1, another transmembrane mucin, has been shown to interact with growth factor receptors – such as the ErbB family members, which are otherwise restricted to the lateral and basal cell borders (Schroeder *et al*, 2001; Scibetta *et al*, 2001; Canbay, 2003). Moreover, the rat homologue of MUC4 (rMUC4/SMC) was also shown to interact with ErbB2/neu in multiple cell types (Carraway and Idris, 2001a; Carraway *et al*, 2001b, 2002a, 2002b). Therefore, our observations on MUC4–HER2 interaction in SKOV3 have a strong functional relevance in ovarian cancer progression. What yet remains to be investigated is the structural basis of this interaction. On the basis of previous findings with rat MUC4, we may speculate a direct interaction of MUC4 with HER2, involving one of the three EGF domains present in the transmembrane (MUC4-*β*) subunit of MUC4. It is also likely that the cytoplasmic tail of MUC4 or the glycans present on MUC4 may indirectly interact with HER2, as has been shown recently for MUC1-EGFR (Ramasamy *et al*, 2007). The observation that HER2 expression is increased in MUC4-transfected SKOV3 cells is consistent with our previous finding where we observed a correlative decrease in HER2 expression in antisense-RNA-induced stable MUC4-knockdown cells (Singh *et al*, 2004). Altogether, our present data and previous observations clearly indicate a role of MUC4 in the regulation of HER2 expression (Singh *et al*, 2004; Chaturvedi *et al*, 2007). Notably, our observations are not consistent with previous findings, where rat SMC, although forming a complex with ErbB2 and inducing its phosphorylation, did not affect ErbB2 expression (Carraway *et al*, 2001b, 2002a, 2002b; Singh *et al*, 2004). Interestingly, no significant changes in the expression of HER2 transcript were observed in MUC4-transfected SKOV3 cells, suggesting that MUC4-mediated HER2 regulation may occur by post-transcriptional mechanism(s). This is consistent with a recently published report by us in pancreatic cancer cells (Chaturvedi *et al*, 2008). In a recent study, MUC1 has been shown to inhibit the degradation of ErbB1/HER1 by regulating the receptor trafficking (Pochampalli *et al*, 2007) and possibly, a similar mechanism may also exist for MUC4-mediated HER2 regulation, which needs to be investigated.

Another important finding of this study was the enhanced cell motility in the MUC4-transfected SKOV3 cells as compared with the vector-transfected controls (Figure 2A). This was also consistent with our previous results, where we observed a three-fold decrease in cell motility upon silencing of MUC4 expression (Singh *et al*, 2004). Although the exact molecular mechanism for MUC4-associated change in cell motility is yet to be deciphered, we predict an important role for augmented HER2 downstream signalling in this process. Exogenous overexpression of HER2 and its activation has been correlated with increased invasiveness and cellular motility (Grothey *et al*, 2000). Studies on rat MUC4 (SMC) have also demonstrated that MUC4-induced HER2/neu activation may be important in tumour growth and metastasis in rat mammary carcinoma cells (Komatsu *et al*, 2001; Carraway *et al*, 2002a). Results from this study have shown an increased activation of FAK in MUC4-transfected SKOV3 cells, whereas the total FAK remained unchanged. Similarly, we also observed an activation of Akt. Previous studies support our observations that HER2 downstream signalling leads to FAK, Akt and ERK activation and is implicated in cell survival and motility (Gabarra-Niecko *et al*, 2003; Mitra and Schlaepfer, 2006). Recent studies demonstrate that FAK is linked to the downregulation of HER2 activity at the cell periphery, which appears to be important for the formation of motile cells (Mitra and Schlaepfer, 2006). Therefore, the activation of FAK may be related to the increased motility of MUC4-transfected SKOV3 cells. Cell movement is typically associated with a coordinated assembly and disassembly of the cortical actin network (Cunningham *et al*, 1991, 1992). For migration, a cell requires the formation of cell membrane protrusions containing actin filaments and a continuous process of actin polymerisation near the leading edges (Feldner and Brandt, 2002). It has been shown that the cytoplasmic tail of MUC1 may affect actin polymerisation and increase motility in cancer cells (Parry *et al*, 1990). In line with this, our data also shows that the actin cytoskeleton is predominantly localised at the leading edges and cellular projections of MUC4-overexpressing SKOV3 cells.

In conclusion, this work is the first report of the direct association of MUC4 with the motile phenotype in ovarian cancer cells. The ectopic expression of MUC4 alters HER2 expression at protein levels, thus potentiate its activation of downstream signalling. Overall, our results demonstrate that MUC4 plays a crucial role in regulating the motility of ovarian cancer cells possibly by altering the expression and activation of HER2 and its downstream signalling.
